# *Arabidopsis* Circadian Clock Repress Phytochrome a Signaling

**DOI:** 10.3389/fpls.2022.809563

**Published:** 2022-05-11

**Authors:** Yang Liu, Yanzhao Sun, Heng Yao, Yanyan Zheng, Shuyuan Cao, Haiyang Wang

**Affiliations:** ^1^College of Horticulture, China Agricultural University, Beijing, China; ^2^State Key Laboratory of Agrobiotechnology, China Agricultural University, Beijing, China; ^3^College of Life Sciences, South China Agricultural University, Guangzhou, China

**Keywords:** FHY3/FAR1, FHY1/FHL, TOC1, CCA1, PHYA, clock

## Abstract

The plants’ internal circadian clock can strongly influence phytochrome signaling in response to the changes in the external light environment. Phytochrome A (phyA) is the photoreceptor that mediates various far-red (FR) light responses. phyA signaling is modulated by FHY3 and FAR1, which directly activate the transcription of FHY1 and FHL, whose products are essential for light-induced phyA nuclear accumulation and subsequent light responses. However, the mechanisms by which the clock regulates phyA signaling are poorly understood. Here, we discovered that FHY1 expression is diurnally regulated, peaking in the middle of the day. Two *Arabidopsis* core clock components, CIRCADIAN CLOCK ASSOCIATED1 (CCA1) and TIMING OF CAB EXPRESSION1 (TOC1), repress FHY3/FAR1-mediated *FHY1/FHL* activation. Consistently, the specific expression pattern of FHY1 under diurnal conditions is altered in *cca1-1*, *toc1-101*, *CCA1*, and *TOC1* overexpression plants. Furthermore, far-red induced gene expression and particularly nuclear accumulation of phyA are compromised in TOC1 and CCA1 overexpression seedlings. Our results therefore revealed a previously unidentified FHY1 expression pattern in diurnal cycles, which is negatively regulated by CCA1 and TOC1.

## Introduction

The circadian clock regulates almost every aspect of metabolism and development in plants. The *Arabidopsis* circadian clock consists of a central oscillator loop that connects morning- and evening-phase circuits (Pruneda-Paz and Kay, 2010). The central loop is composed of three genes, two morning-expressed Myb transcription factors—CIRCADIAN CLOCK ASSOCIATED1 (CCA1) and LATE ELONGATED HYPOCOTYL (LHY) and an evening-expressed pseudoresponse regulator TIMING OF CAB EXPRESSION 1 (TOC1; Alabadí et al., 2001; Kamioka et al., 2016). CCA1/LHY and TOC1 repress each other’s expression, and thus constituting a negative feedback loop (Alabadí et al., 2001; Gendron et al., 2012). Besides the essential role of CCA1/LHY and TOC1 as central oscillators that control the activity of other clock components, this circularity module also regulates diverse output processes, including iron and ROS homeostasis, leaf senescence and photosensory pathway-mediated hypocotyl growth (Lai et al., 2012; Soy et al., 2016; Song et al., 2018; Xu et al., 2019).

Light controls the growth of plants through a network of photoreceptors. *Arabidopsis* has five red/far-red light-absorbing phytochromes (phyA, phyB, phyC, phyD, and phyE), of which phyA and phyB play the most predominant functions (Sharrock and Quail, 1989; Clack et al., 1994). PhyA is the only photoreceptor mediating various plant responses to FR light compared with darkness (Quail et al., 1995). Upon light irradiation, phyA is activated and rapidly translocated into nuclear, representing a critical step of phyA signaling (Kim et al., 2000). Two small plant-specific proteins, FAR-RED ELONGATED HYPOCOTYL1 (FHY1) and its homolog FHY1-LIKE (FHL), are essential for nuclear accumulation of light-activated phyA and subsequent light responses (Hiltbrunner et al., 2005, 2006; Rösler et al., 2007). The activation and repression of FHY1/FHL-phyA signaling are energetically demanding. FHY3 and FAR1, as a new type of transposase-derived transcription factors, activate *FHY1*/*FHL* gene expression directly, which in turn facilitates phyA nuclear accumulation on FR light irradiation (Lin et al., 2007). Mutant seedlings that lack FHY3 and FAR1 displayed elongated hypocotyls and impaired nuclear accumulation of phyA under continuous FR light (Lin et al., 2007). Thus, the regulators which modulate FHY3 and FAR1 activity inevitably affect the FHY1/FHL levels and ultimately the phyA signaling pathway. The function of the bZIP transcription factor ELONGATED HYPOCOTYL5 (HY5) as a repressor for *FHY1*/*FHL* expression has been reported. HY5 negatively regulates *FHY1*/*FHL* expression *via* physical interaction with FHY3/FAR1 and interferes with their binding to the *FHY1*/*FHL* promoters (Li et al., 2010). Recently, the JA (jasmonic acid) signaling repressor JAZ1 was reported to interact with FHY3 and FAR1, and inhibit their transcriptional activity on *FHY1*/*FHL* expression (Liu et al., 2020a). The antagonistic interaction of FHY3/FAR1 with HY5 and JAZ1 may provide a mechanism for fine-tuning the phyA signaling pathway by light and hormone, respectively.

Daily changes of light, defining the diurnal cycle of everyday, are an essential input to the circadian clock. In plants, the photoreceptor phytochrome and cryptochrome set the clock by transducing the light signal to the central oscillator, which is called the input pathway. For instance, phyA and phyB are responsible for light-mediated entrainment of the circadian clock under FR and R radiation, respectively (Somers et al., 1998). On the contrary, like other clock-controlled output traits, the process of light input is rhythmic and regulated by the clock machine. Through a gating mechanism, the circadian clock modulates the light responsiveness of physiological outputs at different times of the day. For example, expression of the CAB genes that encode the chlorophyll a/b-binding proteins is not only induced by light but also controlled by the circadian rhythm, suggesting that the clock modulates the acute response to light (Millar and Kay, 1996). In addition, some key clock components, like PRR7, PRR9 and ELF3, are potentially involved in light input to the clock (McWatters et al., 2000; Farre et al., 2005). Despite this progress, how circadian clock components modulate phytochrome activity remains to be answered. A previous study has revealed that the action of the phyA signaling pathway is regulated at multiple levels. For example, the transcription level of *PHYA* is regulated by the circadian clock with peaking in the late afternoon (Hall et al., 2001). Importantly, under daily photoperiods of far-red light, the number of nuclei with phyA speckles is higher during daytime than during the night (Kircher et al., 2002), but the underlying mechanism remains obscure.

Here, our study revealed that the central oscillator components, CCA1 and TOC1, could suppress the function of FHY3/FAR1, which in turn inactivates *FHY1*/*FHL* expression and nuclear accumulation of phyA. Furthermore, we found that the action of CCA1 and TOC1 conferred the circadian expression pattern of FHY1, which might provide an adaptive mechanism for plant perception of far-red light under diurnal cycles.

## Materials and Methods

### Plant Materials and Growth Conditions

The wild type *Arabidopsis thaliana* plants used in this study are of the Col-0 ecotype unless otherwise indicated. The *fhy3-1*, *far1-2*, *fhy3-1 far1-2*, *35S::Flag-FHY3-HA* and *35S::FHY1-GFP* have been described previously (Lin et al., 2007; Li et al., 2011; Chen et al., 2012). The *cca1-1* and *CCA1-OX* were in Wassilewskija-2 ecotype (Wang and Tobin, 1998; Green and Tobin, 1999). *35S::FLAG-CCA1-HA* and *35S::FLAG-TOC1-HA* (*TOC1-OX*) were described previously (Li et al., 2011). Plants were grown on MS containing 2% sucrose and 0.75% agar under 12-h light/dark conditions (75 μmol m^−2^ s^−1^) in a Percival growth chamber (Percival Scientific).

### Plasmid Construction

To generate *FHY1p:LUC* transgenic plants, the amplified FHY1 promoter was subcloned into the pPZP221-ELF4:LUC vector (Li et al., 2011) through PstI/BamHI sites.

### Yeast Assays

Yeast one-hybrid assays were performed as described previously (Li et al., 2010).

### Gene Expression Analysis

Total RNA was extracted from seedlings using Trizol (Invitrogen). The first-strand cDNA was synthesized from 1 μg of RNA using reverse transcriptase (Tiangen). The cDNA was diluted 1:10 and subjected to quantitative PCR using SuperReal PreMix Plus (Tiangen) and a 7,500 Real-Time PCR System (Applied Bio-systems) cycler. Gene expression levels were normalized to *PP2A* and are shown relative to the expression levels in wild type. Primers are listed in Supplementary Table 1.

### Transient Expression Assay

Transient expression assays were performed as described previously (Li et al., 2011). The reporter and effector constructs were transformed into Agrobacterium strain EHA105. The Agrobacterium solutions containing the reporter or effector constructs were coincubated for 2 h and infiltrated into 3–4-week-old *N. benthamiana* leaves. Plants were incubated under continuous white light for 3 d after infiltration. The firefly LUC activity was photographed after spraying with 1 mM luciferin (Goldbio). For the dual-luciferase quantification assay, firefly luciferase and Renilla luciferase activities were assayed as described previously (Li et al., 2010).

### Western Blot Analysis

For anti-FHY1 immunoblots, *Arabidopsis* seedlings were ground to a fine powder and resuspended in 200–500 μl of Lysis Buffer (50 mm Tris–HCl, pH 7.5, 150 mm NaCl, 10 mm MgCl_2_, 0.1% Tween 20, 1 mm PMSF, 40 mm MG132, and 1X complete protease inhibitor cocktail). Sample loading was made with 5X Laemmli’s buffer in 10% SDS–polyacrylamide gel electrophoresis gels. According to the manufacturer’s recommendations after transference to PVDF membranes, the immunoblotting was detected with anti-GFP (1:1000) antibody (MBL; 598–7). For CCA1 and TOC1 immunoblots, proteins were detected with anti-FLAG (1:4000) antibody (MBL; M185-7). The secondary antibody used is HRP-conjugate (MBL; 1:8000).

### Fluorescence Microscopy

For fluorescence microscopy analyses, seedlings were grown on MS medium for 5 d and then released to far-red light for 10 h. At least 10 independent lines for each cross combination were examined using a Zeiss LSM 510 multiphoton microscope.

## Results

### *FHY1* Gene Is Clock Regulated

**Figure 1:**
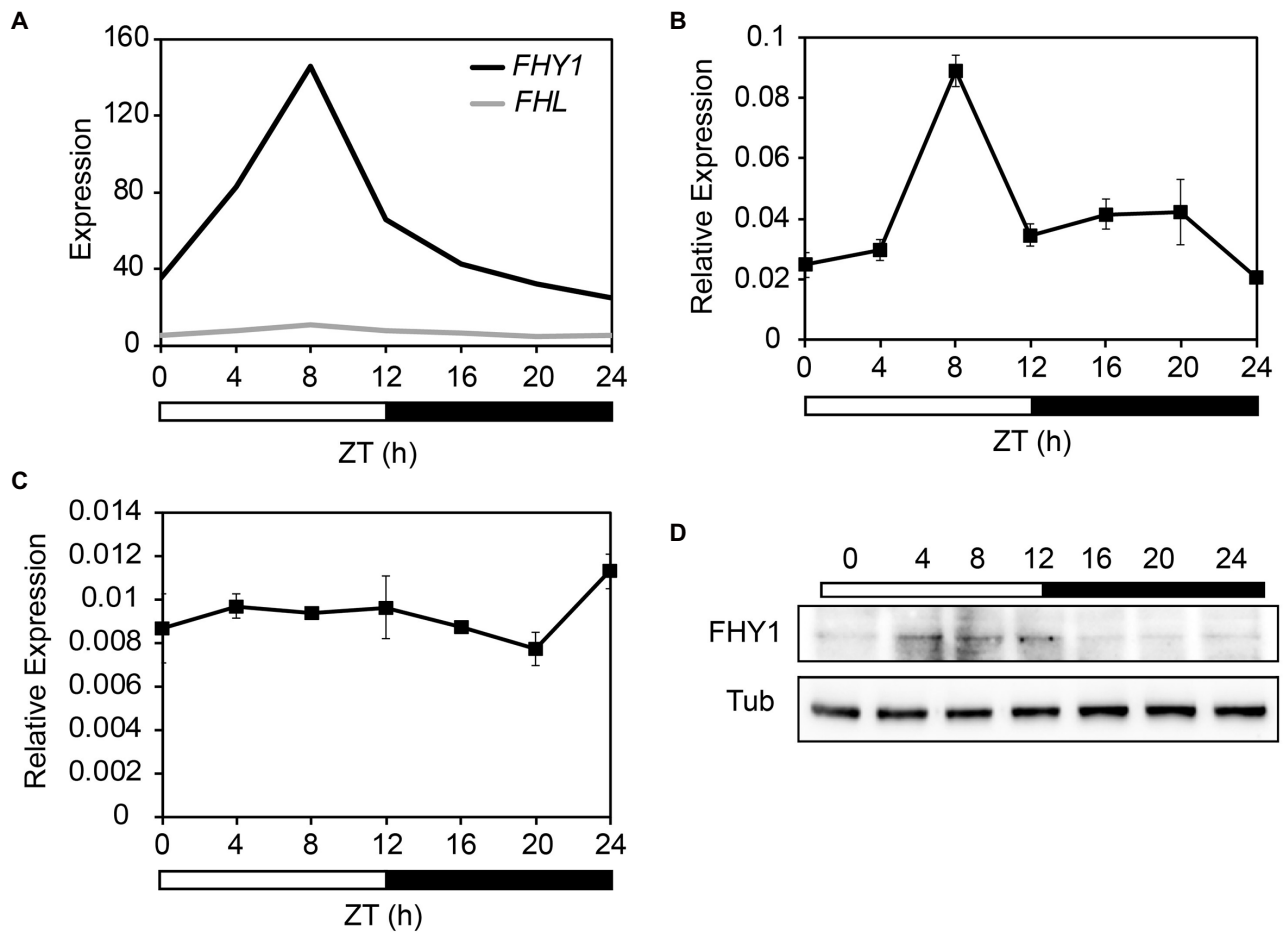
FHY1 encodes a middle-day-phased protein. **(A)** Time-course gcRMA (GeneChip Robust Multiarray Averaging) values of FHY1 and FHL expression (from Diurnal database, http://diurnal.mocklerlab.org/ Mockler et al., 2007) under 12 l:12D condition. **(B,C)** qRT-PCR analysis of *FHY1* **(B)** and *FHL* **(C)** expression in wild-type (Col-0) seedlings grown in diurnal cycles. Values are means ± SD; *n* = 3. **(D)** Immunoblot assay shows that FHY1 protein level oscillates in the diurnal cycle. Tubulin (Tub) was used as an internal control. 5-d-old, 12 l:12D entrained FHY1-GFP seedlings were harvested at the indicated time points. Anti-GFP antibodies (1:4000; MBL) were used to detect FHY1 protein.

To investigate whether the expression levels of *FHY1* and *FHL* exhibit circadian rhythm, we first investigated these two genes in the public DIURNAL database (Mockler et al., 2007; Michael et al., 2008). Analysis of the microarray data from 12 l:12D time courses (Light:Dark hours = 12:12) revealed that expression of *FHY1* oscillated rhythmically, with a peak of expression occurring at Zeitgeber time 8 (ZT8; Figure 1A). FHL, the close homolog of FHY1, lacked such rhythmical expression and kept steady around the whole day. Furthermore, the expression pattern of *FHY1* and *FHL* was confirmed by quantitative PCR (qPCR) analysis. Similarly, *FHY1* mRNA accumulated after dawn, reached a maximum at ZT8 and subsequently decreased toward the end of night (Figure 1B). Moreover, expression of *FHL* is not robustly regulated by the circadian clock (Figure 1C). To further confirm the circadian expression pattern of *FHY1*, we generated *FHY1p:LUC* transgenic line and examined its luciferase activity under continuous light conditions. As expected, the result is consistent with our qRT-PCR assay with FHY1 expression peak around ZT8 (Supplementary Figure 1). As FHY3 is essential for *FHY1* gene expression, we tested the role of FHY3 on *FHY1* rhythm and found that the circadian rhythm of *FHY1* is lost in *fhy3-4* mutant, while enhanced in the FHY3 overexpression line (Supplementary Figure 2). To monitor whether FHY1 protein levels oscillate, the transgenic line expressing FHY1-GFP under the control of CaMV 35S promoter (35S::FHY1-GFP) was used to detect the FHY1 protein abundance under diurnal cycles. The results showed that FHY1-GFP fusion protein accumulates after dawn, reaching a peak from mid-day to the afternoon (ZT4-ZT12) and a trough through the whole night (Figure 1D).

**Figure 2:**
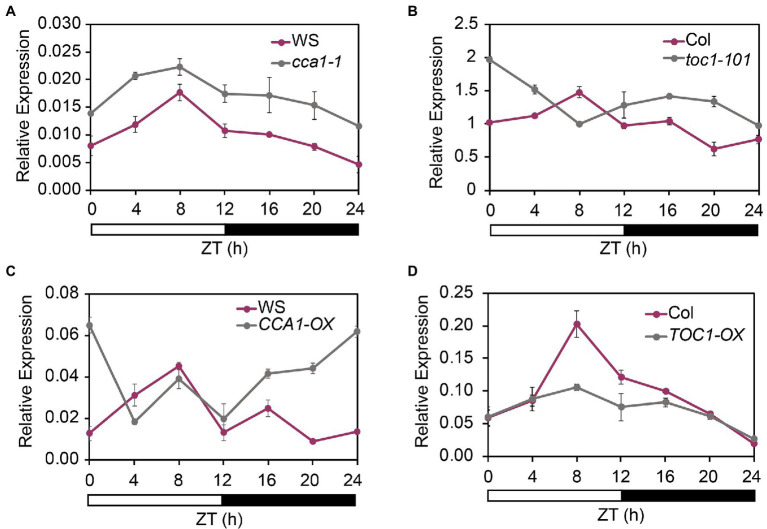
CCA1 and TOC1 regulate the rhythmic expression of *FHY1*. **(A,B)** qRT-PCR analysis of *FHY1* expression in wild type (Col-0) and *cca1-1* **(A)**, and *toc1-101* **(B)** mutant seedlings grown in diurnal cycles. Values are means ± SD; *n* = 3. **(C)** and **(D)** qRT-PCR analysis of *FHY1* expression in wild type (WS) and *CCA1-OX* **(A)**, and *TOC1-OX* **(B)** seedlings grown in diurnal cycles. Values are means ± SD; *n* = 3.

To determine whether the cycling expression pattern of *FHY1* was affected by the core clock components, we examined the *FHY1* expression pattern in TOC1 and CCA1 overexpression and mutant lines (*TOC1-OX*, *CCA1-OX*, *toc1-101*, and *cca1-1*) under diurnal cycles. The results showed that the circadian expression pattern of *FHY1* changed in these lines compared with wild type. In *cca1-1* and *toc1-101* mutants, the *FHY1* level increased compared with wild type (Figures 2A,B). In CCA1-OX transgenic plants, *FHY1* expression increased at night, and the peak shifted to dawn (Figure 2C). In addition, the *FHY1* peak in *TOC1-OX* at ZT8 disappeared and the circadian pattern was absent (Figure 2D). These results suggested that CCA1 and TOC1 affect *FHY1* expression and shape the precise middle-day-phased expression pattern of *FHY1*.

Given that FHY1-mediated phyA nuclear accumulation is essential for phyA signaling, it is interesting to test whether genes involved in phyA signaling are also clock regulated as well as FHY1. Thus, we obtained time-course expression profiles of 224 phyA-induced genes (selected from Chen et al., 2014) from the DIURNAL database (Supplementary Dataset S1). Strikingly, we found that a vast majority of these genes display time-of-day specific phases under diurnal conditions. As expected, large numbers of genes peaked at noon (ZT8), coinciding with the rhythm of *FHY1* expression (Supplementary Figure 3). These findings indicated that phyA signaling pathway is regulated by the clock.

### CCA1 and TOC1 Repress Transcriptional Activation Activity of FHY3

**Figure 3:**
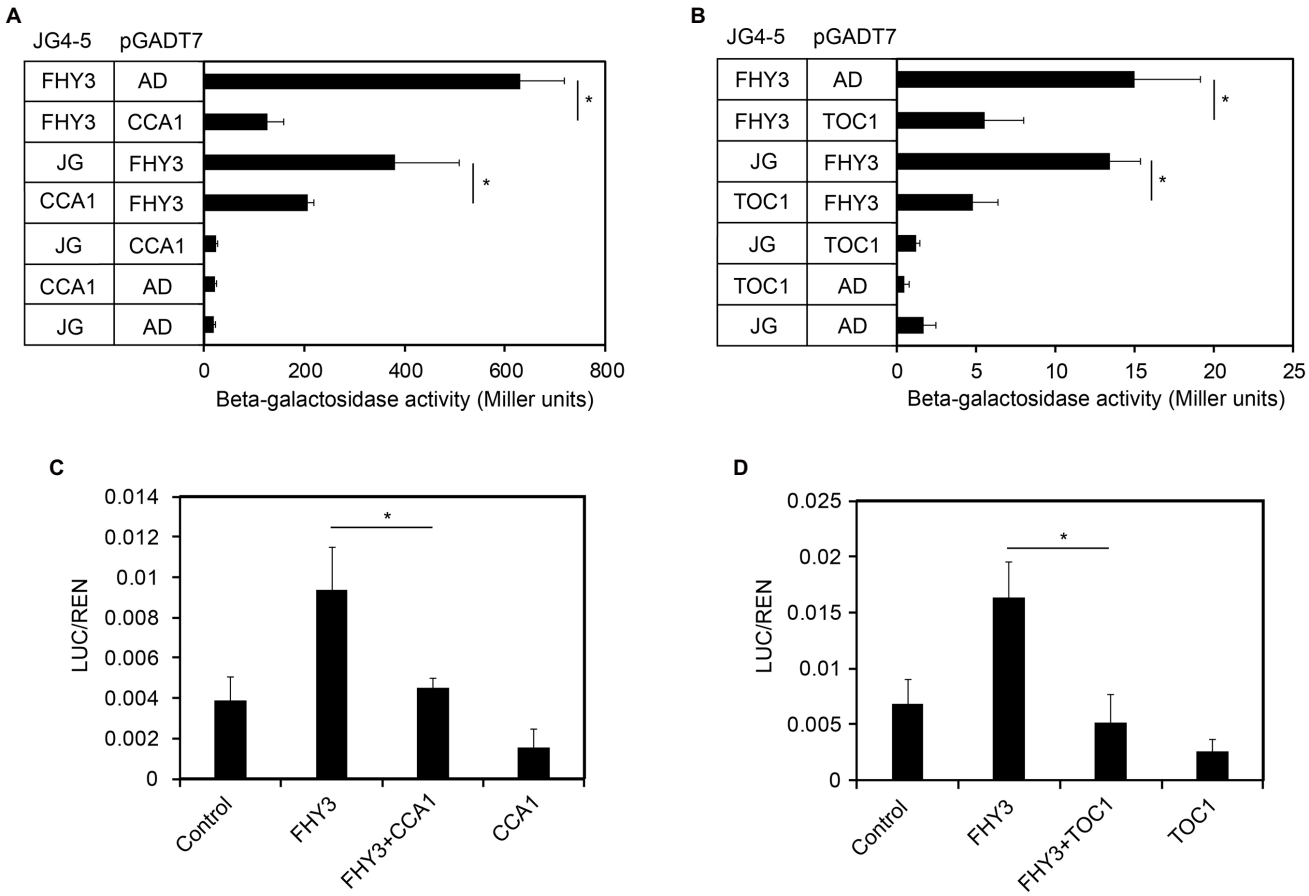
CCA1 and TOC1 negatively regulate FHY3 activated FHY1 transcription in yeast and plant cells. **(A, B)** Quantification of β-galactosidase activity in yeast cells harboring the FHY1p-B:LacZ reporter construct and coexpressing FHY3 and CCA1 **(A)**, FHY3 and TOC1 **(B)** protein combinations. Error bars represent SD (*n* = 6). **(C, D)** Relative reporter activity in tobacco cells transiently transformed with the indicated effector and reporter constructs. FHY3, CCA1 and TOC1 are expressed by the 35S:FHY3, 35S:CCA1 and 35S:TOC1 effector plasmids (see “Materials and Methods”). Tobacco leaves were kept in white light for 4 d after infiltration. The relative LUC activities normalized to the REN activity are shown (LUC/REN). Error bars represent SD (*n* = 3).

A previous study showed that CCA1 could directly interacts with FHY3/FAR1and represses their transcriptional activating activity (Li et al., 2011). Recently, we have confirmed the interactions between FHY3/FAR1 and TOC1 (Liu and Wang, 2020b). Therefore, we speculated that CCA1 and TOC1 might affect FHY3/FAR1-mediated *FHY1* transcription. To begin to assess potential CCA1 and TOC1 repression of FHY3 activity, we conducted the modified yeast one-hybrid assay to test the ability of FHY3 to promote *FHY1* expression when CCA1 or TOC1 was introduced. As expected, our results showed that FHY3 could activate *FHY1p:LacZ* reporter gene expression. In contrast, the inclusion of CCA1 or TOC1 removed FHY3’s activation activity, suggesting that CCA1 and TOC1 negatively regulate FHY3/FAR1-activated *FHY1* expression (Figures 3A,B). In parallel, we performed a transient gene expression assay in *N. benthamiana* leaf to test the effect of FHY3-CCA1 and FHY3-TOC1 interaction on *FHY1* expression. Consistent with the results of Y1H assay, FHY3 could effectively activate the *FHY1p:LUC* reporter gene expression, whereas co-expression of CCA1 or TOC1 with FHY3 significantly repressed the expression of the *FHY1p:LUC* reporter (Figures 3C,D), indicating that both CCA1 and TOC1 can suppress the transcriptional activation activity of FHY3 on *FHY1* transcription *in planta*.

### CCA1 and TOC1 Repress Expression of *FHY1* and *FHL*

**Figure 4:**
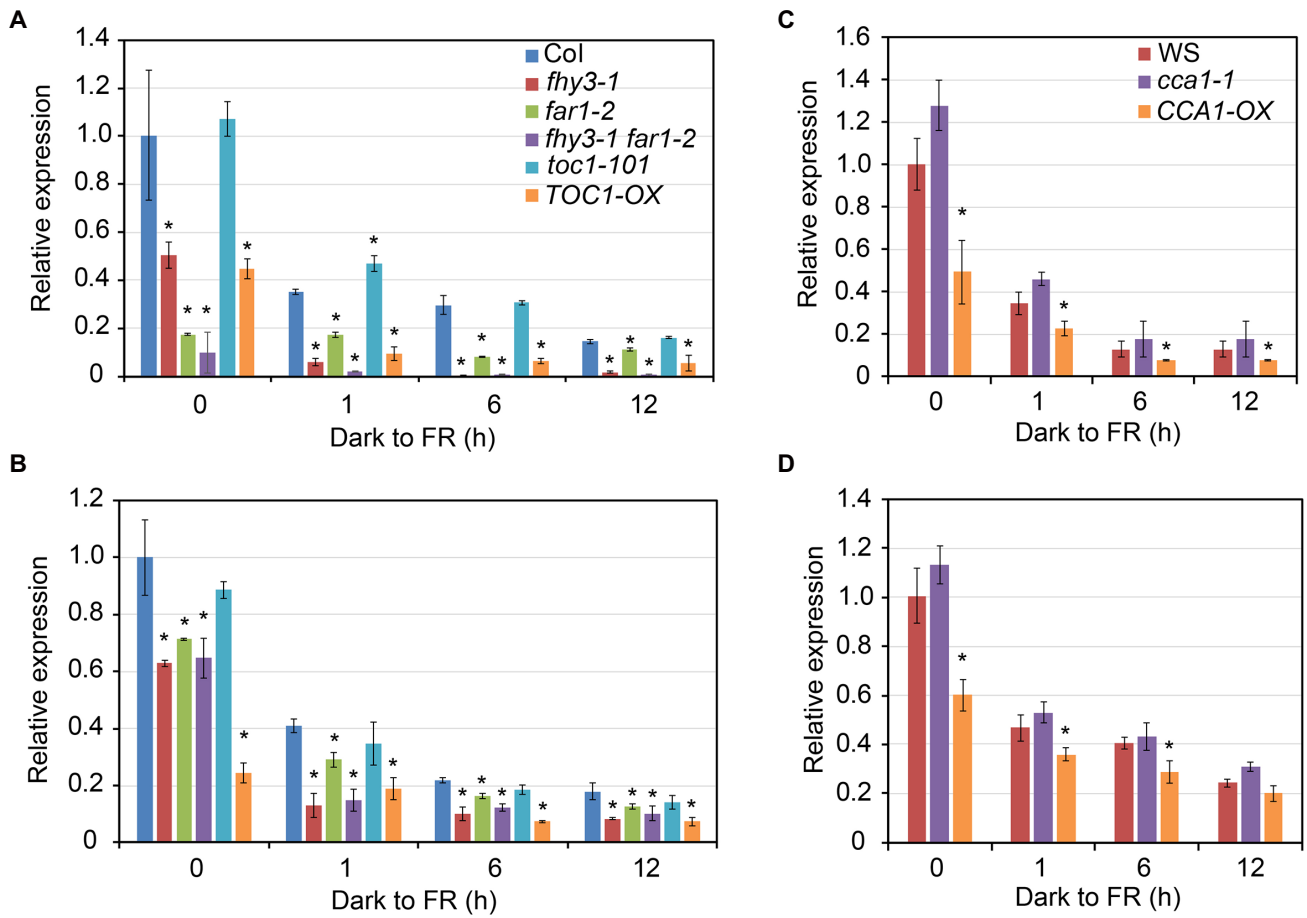
CCA1 and TOC1 negatively regulate FHY1 and FHL expression. **(A, B)** qRT-PCR analysis of *FHY1* **(A)** and *FHL* **(B)** expression in wild type (Col-0), *fhy3-1*, *far1-2*, *fhy3-1 far1-2*, *toc1-101*, and *35S:TOC1* seedlings. **(C, D)** qRT-PCR analysis of *FHY1* **(C)** and *FHL* **(D)** expression in wild type (WS), *cca1-1* and *CCA1ox* seedlings. Seedlings are grown in darkness for 4 d and then transferred to FR light for various time periods. Asterisks indicate significant differences from wild type plants (*p* < 0.05, Student’s *t*-test). Values are means ± SD; *n* = 3.

To investigate the role of CCA1 and TOC1 in regulating *FHY1* expression *in vivo*, we examined *FHY1* expression level in *toc1-101*, *cca1-1* mutants and the overexpression lines of *TOC1-OX* and *CCA1-OX*, compared with the wild type (Col-0 and Ws ecotypes) and *fhy3-1*, *far1-2*, and *fhy3-1 far1-2* mutants. Previous studies showed that *FHY1* and *FHL* transcript levels displayed a declined expression pattern when dark-grown seedlings were transferred to far-red light (Lin et al., 2007; Li et al., 2010). qRT-PCR analysis revealed that *FHY1* and *FHL* transcript levels were significantly reduced in the *CCA1-OX* and *TOC1-OX* plants, similar to the mutants of *fhy3-1*, *far1-2* and *fhy3-1 far1-2* (Figures 4A–D). In contrast, the *FHY1* and *FHL* expression in *cca1-1* and *toc1-101* mutants were not significantly altered compared with wild-type plants.

To determine whether far-red light affects the activity of CCA1 and TOC1, we then examined the mRNA and protein levels of CCA1 and TOC1 in this time course. We found that, when dark-grown seedlings were exposed to far-red light, both the CCA1 mRNA and protein levels started to decrease, while TOC1 showed an increased pattern (Supplementary Figures 4, 5).

**Figure 5:**
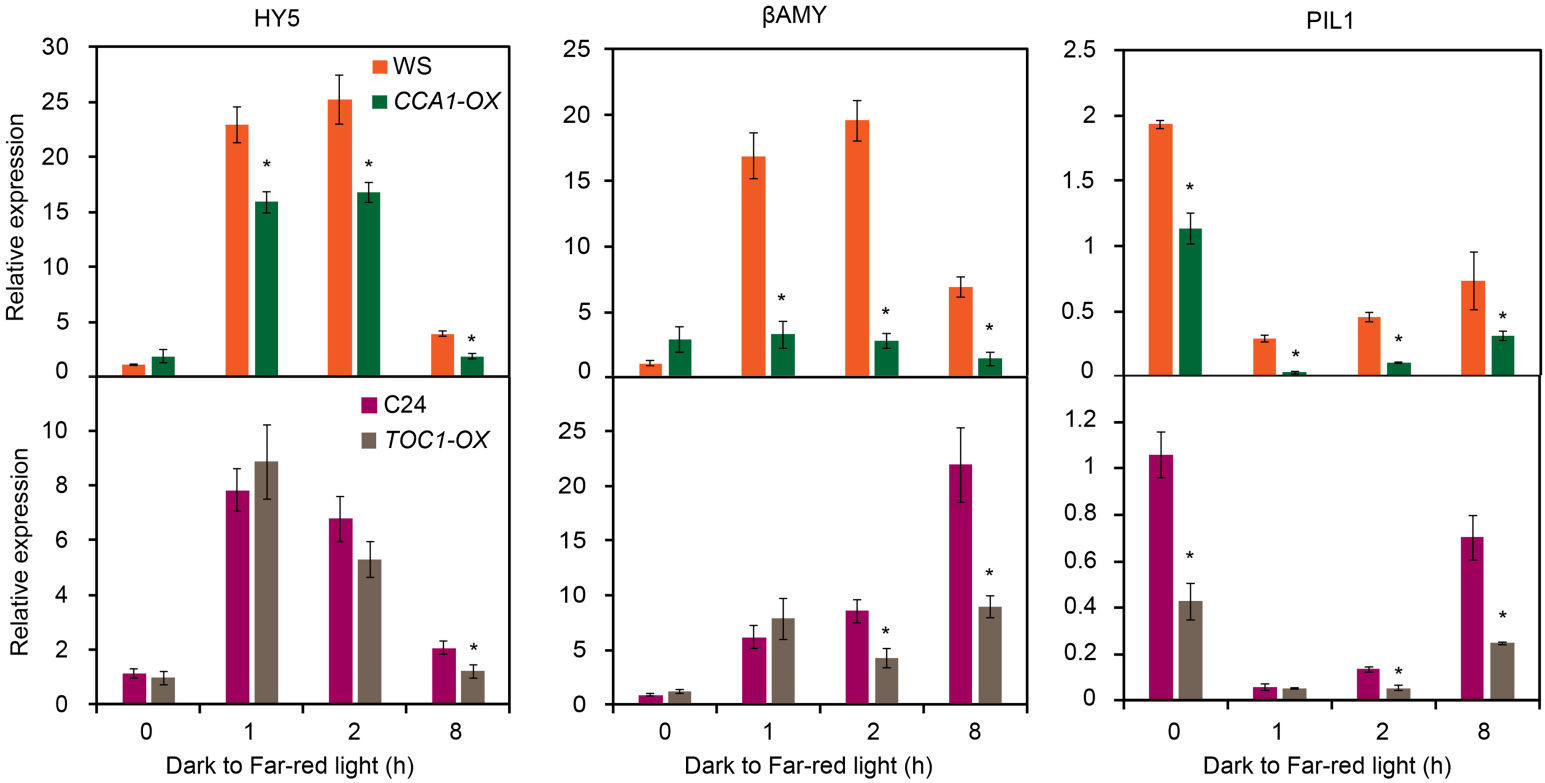
Expression of FR responsive genes is reduced in *CCA1-OX* and *TOC1-OX* seedlings. qRT-PCR analysis of *HY5*, *βAMY*, *PIL1*, *CAB2*, *CAB3* and *HFR1* expression in wild type and CCA1-OX (upper panel), and TOC1-OX (lower panel) seedlings grown in darkness for 4 d and then transferred to FR light for various time periods. Asterisks indicate significant differences from wild type plants (*p* < 0.05, Student’s *t*-test). Values are means ± SD; *n* = 3.

Given FHY1 and FHL are essential for phyA nuclear accumulation and subsequent far-red light signaling, we hypothesized that the expression of FR responsive genes in *CCA1-OX* and *TOC1-OX* transgenic plants might be compromised. To this end, we examined the expression of six FR responsive genes (*HY5*, *β-AMY*, *PIL1*, *CAB2*, *CAB3* and *HFR1*) in *TCO1-OX* and *CCA1-OX* plants grown under dark to FR conditions. As shown in Figure 5, expression levels of these six FR responsive genes significantly declined in *CCA1-OX* and *TOC1-OX* plants compared with the wild type in some time points. Moreover, we tested the expression levels of these six genes in *toc1-101* and *cca1-1* mutants. Similar to the results of FHY1 expression, expression of these six genes did not differ dramatically from the wild type (Supplementary Figure 6). Together, these data suggested that CCA1 and TOC1 antagonize FHY3-mediated *FHY1* expression and FR responsive gene expression.

Furthermore, we examined the phenotype of hypocotyl growth under continuous FR light conditions. The results showed that *cca1-1* mutant displayed short hypocotyl, while CCA1-OX plant displayed long hypocotyl, which is consistent with the expression of FR responsive genes (Supplementary Figure 7A). However, hypocotyl of *toc1* mutant seemed longer than wild type, and no noticeable difference was observed between TOC1-OX and wild-type plants, implying other unknown mechanisms existed in TOC1-mediated hypocotyl growth in FR light conditions (Supplementary Figure 7B).

### Nuclear Localization of phyA Is Inhibited in TOC1-OX and CCA1-OX Plants

**Figure 6:**
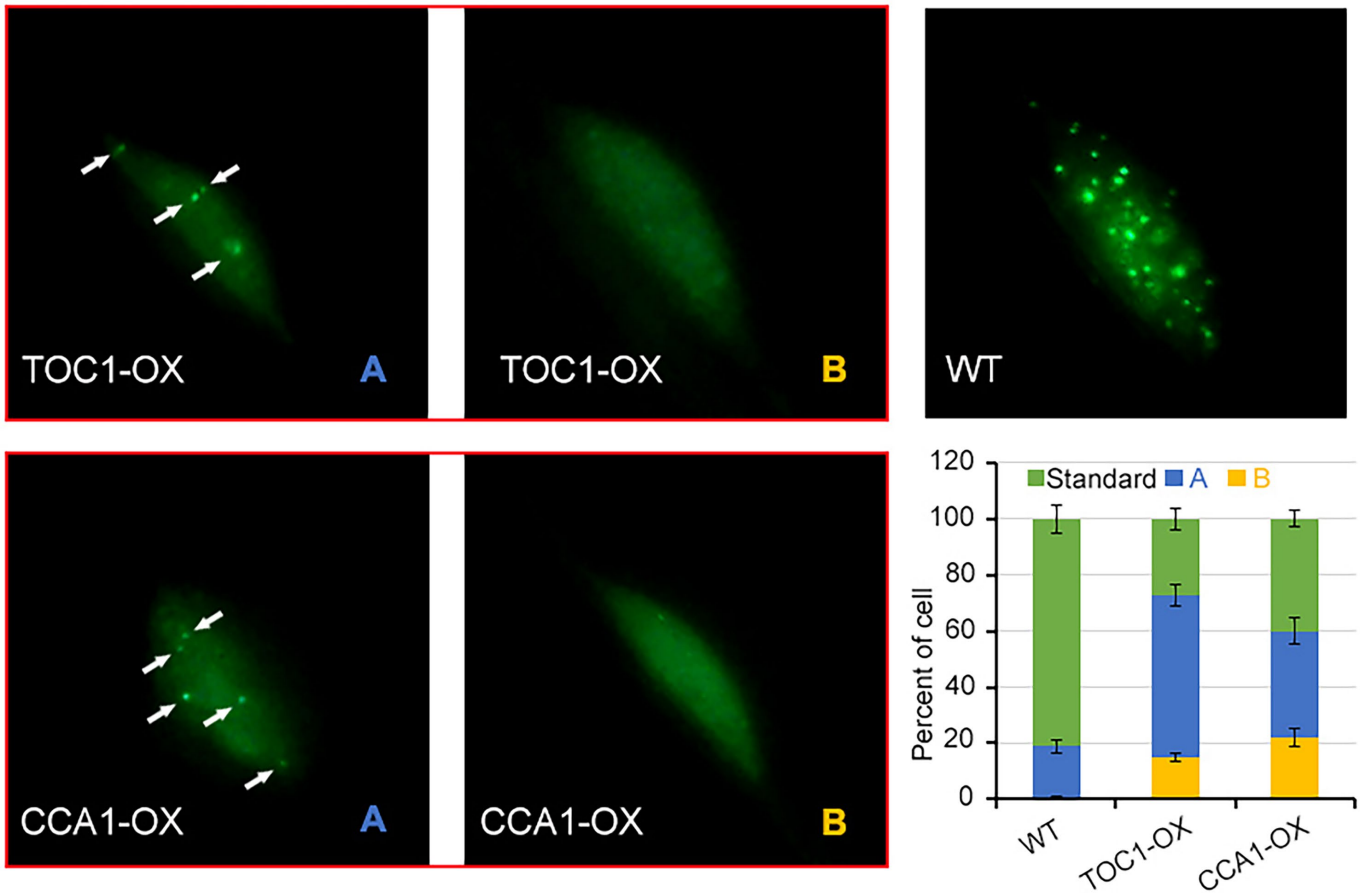
The nuclear localization of phyA is inhibited in TOC1-OX and CCA1-OX plants. 4-d-old dark-grown *phyA-GFP*, *TOC1-OX phyA-GFP* and *CCA1-OX phyA-GFP* seedlings were transferred to FR for 8 h before detecting the phyA nuclear accumulation. For each genotype, at least 20 individual lines were observed, and different types of phyA localization were counted.

To gain insight into the mechanism by which CCA1 and TOC1 antagonize phyA signaling, we analyzed the activity of phyA accumulation into nuclear upon FR irradiation. We generated phyA-GFP/TOC1-OX and phyA-GFP/CCA1-OX plants by crossing. In dark-grown seedlings, phyA-GFP was homogeneously dispersed in the cytoplasm as previously described (Kircher et al., 1999; Hisada et al., 2000). Strikingly, when dark-grown seedlings were transferred into far-red light for 8 h, nuclear accumulation of phyA-GFP was significantly reduced in TOC1-OX and CCA1-OX plants compared with wild-type phyA-GFP seedlings (Figure 6). We divided the status of phyA-GFP nuclear accumulation into three types: standard (like wild type, more photobodies); A, few photobodies; and B, no photobodies. Quantitative analysis of these three types revealed that, in TOC1-OX and CCA1-OX plants, standard types are prominently reduced (only 29% in TOC1-OX; 40% in CCA1-OX), abnormal type A (56% in TOC1-OX; 38% in CCA1-OX) and type B (15% in TOC1-OX; 22% in CCA1-OX) appear and increase compared with wild type. These findings suggested that TOC1 and CCA1 repress phyA mediated FR signaling pathway *via* downregulation of *FHY1* level and subsequent phyA nuclear accumulation.

## Discussion

**Figure 7:**
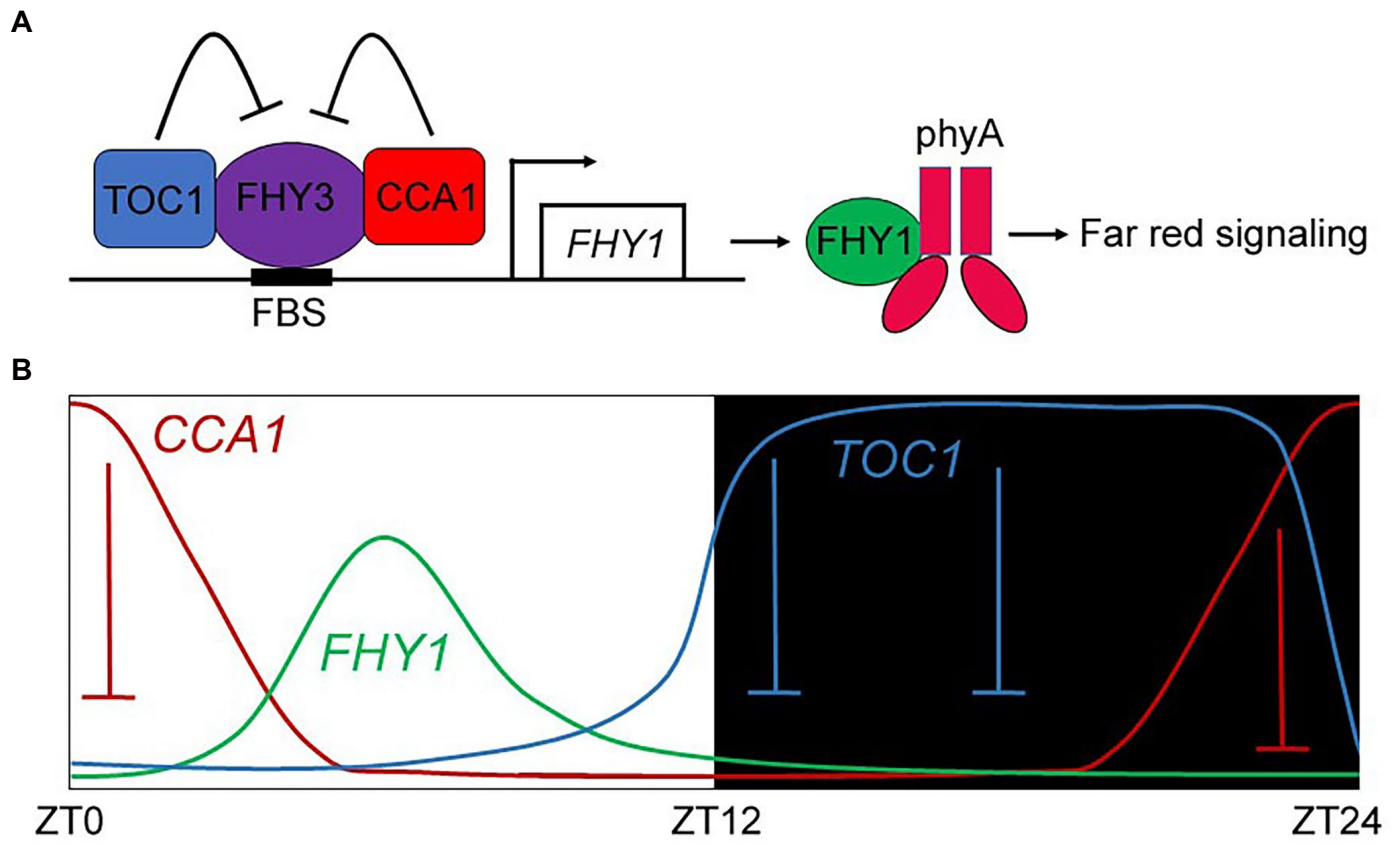
A proposed model depicting the repression effect of TOC1 and CCA1 on *FHY1* transcription and contributing to its circadian expression pattern. **(A)** TOC1 and CCA1 repress the FHY3 transcriptional activity on *FHY1*, which impaired the nuclear transport of phyA and downstream FR signaling. **(B)** In diurnal cycles, CCA1 and TOC1 repressed *FHY1* expression levels in the morning and evening, resulting in peaked expression of *FHY1* in the noon.

In this study, we revealed a previously unidentified *FHY1* expression pattern in diurnal conditions. The clock components TOC1 and CCA1 modulated *FHY1* expression and conferred its circadian rhythm with peaking at the mid-day. Furthermore, we presented evidence to show that TOC1 and CCA1 inactivate phyA signaling *via* repressing FHY3/FAR1-activated *FHY1* and *FHL* transcription. Given the reported interactions of FHY3-CCA1 and FHY3-TOC1 (Li et al., 2011; Liu and Wang, 2020b), we proposed a model in which CCA1 and TOC1 act as transcriptional repressors of FHY3, thereby reducing the *FHY1* transcription level and dysfunction of phyA nuclear accumulation (Figure 7). In addition, CCA1 and TOC1 also limit and shape the *FHY1* expression pattern under diurnal conditions (Figure 7B).

Besides CCA1 and TOC1, the bZIP transcription factor HY5 has been reported to repress FHY3-activated *FHY1* transcription (Li et al., 2010). Unlike CCA1 and TOC1, HY5 can directly bind the ACE element in the *FHY1* promoter. Due to the close location of FHY3 and HY5 binding sites on *FHY1* promoter, HY5 interacts and interferes with FHY3 for binding to *FHY1* promoter (Li et al., 2010). In addition, CCA1 can physically interacts with HY5, and they act synergistically on circadian genes expression (Andronis et al., 2008). Therefore, the role of HY5 in the clock-mediated *FHY1* regulation will be interesting to investigate in future studies.

We demonstrate that the protein and mRNA accumulation of *FHY1* followed a diurnal rhythm and exhibited maximum expression in the light phase (around ZT8). It has been reported that the circadian clock regulates promoter activity and/or mRNA accumulation of PHY and CRY genes (Tóth et al., 2001). Among them, phyA promoter reporter activity and phyA mRNA displayed a biphasic curve, with the first peak appearing just after the lights-on signal, which is very similar to *FHY1* expression. Thus, the inner coincidence of the photoreceptor phyA with the transfer conductor FHY1 might be critical for the effective transduction of far-red signaling. Because of this, the phyA signaling downstream genes (phyA-induced) showed a significant oscillation pattern similar to *FHY1* and *phyA* (Supplementary Figure 3).

As the core components of the circadian clock oscillator, evening gene TOC1 and morning CCA1 reciprocally repress each other in the clock network (Alabadí et al., 2001; Gendron et al., 2012). Actually, TOC1 and CCA1 do not always act oppositely in regulating the clock-output pathways. In some cases, they may play the same role. For example, both TOC1 and CCA1 can repress the flowering time (Niwa et al., 2007). In this study, we revealed another case in which both TOC1 and CCA1 acted negatively in regulating the phyA signaling pathway. Our results showed that both TOC1 and CCA1 were implicated in repressing FR signaling pathway *via* inhibiting FHY3-mediated *FHY1* activation. It was noted that the hypocotyl growth of *cca1* mutant and *CCA1OX* plant in FR light conditions is consistent with the repression role of CCA1 on *FHY1* expression (Supplementary Figure 7A), while the hypocotyl growth of *toc1* mutant and *TOC1OX* plant seemed opposite with the molecular evidence of TOC1 (Supplementary Figure 7B). The hypocotyl growth is mainly controlled by the level of PIFs (Soy et al., 2012). Due to the direct repression of PIF3 by TOC1, the *TOC1OX* has a low amount of PIF3 and exhibits short hypocotyl (Soy et al., 2016). Thus, we speculated that, although TOC1OX lead to reduced activity of PIF3 and FHY1 simultaneously, the dominant role of PIF3 in hypocotyl growth may mask the effect of FHY1 under FR light, thus leading to short hypocotyl phenotype in TOC1OX plant. In addition, the inconsistent circumstances of phenotype and gene expression have been described in TOC1-mediated flowering time regulation. The flowering repressor *ELF4* is repressed by TOC1, yet inactivation of TOC1 displays early flowering time, similar to *elf4* mutant (Kikis et al., 2005; Niwa et al., 2007). Thus, as a strong repressor that targets various important genes and pathways, TOC1-related phenotype analysis is complex and requires further attention.

In this study, we presented the first evidence that core clock components control photoreceptor nuclear accumulation. The light-induced phyA nuclear accumulation was impaired in CCA1-OX and TOC1-OX, indicating that the circadian clock regulates light signal input into plant organisms. To confer a selective advantage upon the organism, entrainment must be adaptable. Light signals, especially photoperiod, change with the seasons in temperate latitudes: the optimal phase for a rhythmic process may vary in parallel. Therefore, the activity of the photoreceptor altered by the entrained circadian clock inside the plant organism is vital for adaptation to the outer changing environment.

## Data Availability Statement

The raw data supporting the conclusions of this article will be made available by the authors, without undue reservation.

## Author Contributions

YL and HW designed research. YL, YS, HY, YZ, and SC performed experiments. YL analyzed the data and wrote the article. All authors contributed to the article and approved the submitted version.

## Funding

This work was supported by grants from the National Natural Science Foundation of China (31500239), Chinese Universities Scientific Fund (15052004) and the 315 Talent Program of China Agricultural University.

## Conflict of Interest

The authors declare that the research was conducted in the absence of any commercial or financial relationships that could be construed as a potential conflict of interest.

## Publisher’s Note

All claims expressed in this article are solely those of the authors and do not necessarily represent those of their affiliated organizations, or those of the publisher, the editors and the reviewers. Any product that may be evaluated in this article, or claim that may be made by its manufacturer, is not guaranteed or endorsed by the publisher.

## Supplementary Material

The Supplementary Material for this article can be found online at: https://www.frontiersin.org/articles/10.3389/fpls.2022.809563/full#supplementary-material
