# Supplementary material for: Arabidopsis Circadian Clock Repress Phytochrome a Signaling
Source: Front Plant Sci. 2022 May 11;13:809563. doi: 10.3389/fpls.2022.809563 (PMC9131076; doi:10.3389/fpls.2022.809563)
Supplement: Supplementary file 2 [file Data_Sheet_2.docx]

**Supplementary Figures and Tables**

**
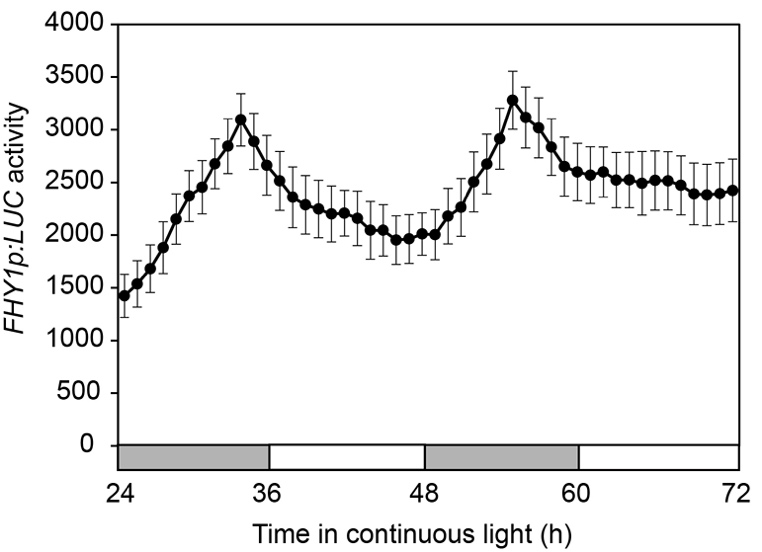
**

Supplementary Figure 1. ﻿Bioluminescence assays showing expression of the *FHY1p:LUC* reporter in wild-type plants. ﻿Seedlings carrying the *FHY1p:LUC* reporter were grown under 12L:12D conditions for 6 d before being transferred to continuous white light. Values are means ± SD (n = 3 technical replicates).


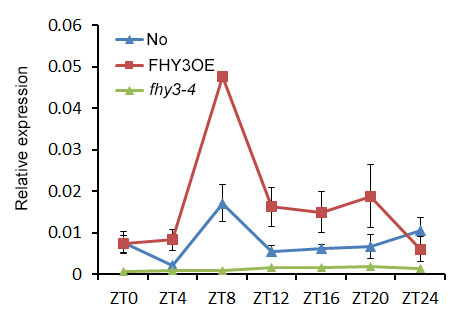


Supplementary Figure 2. The rhythmic expression of *FHY1* in No, FHY3OE (*35S::Flag-FHY3-HA*), and *fhy3-4* mutant. (A) and (B) Values are means ± SD; n=3.


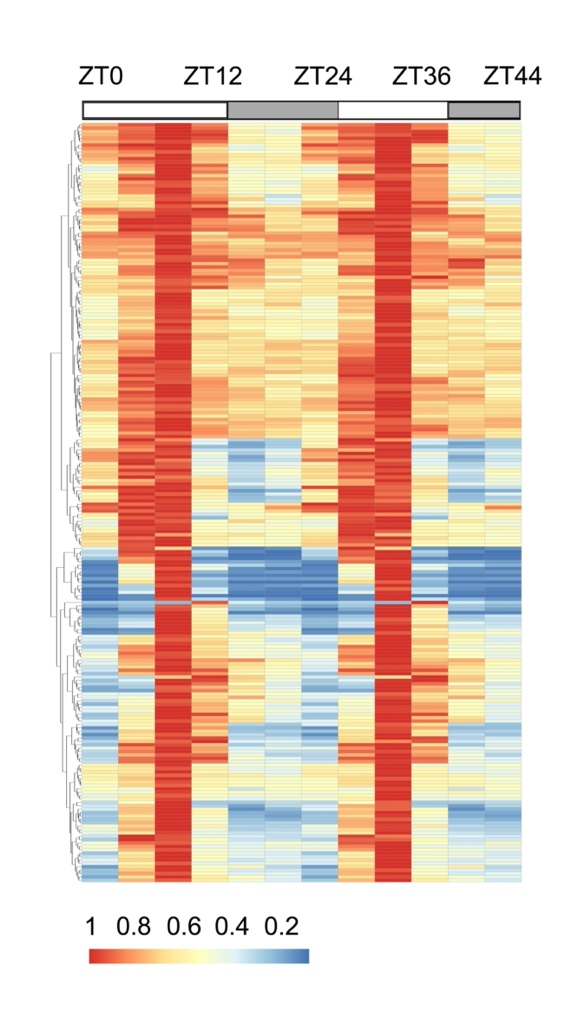


Supplementary Figure 3. Expression profiles of 224 phyA-induced genes transcripts. Expression values were from Diurnal database (<http://diurnal.mocklerlab.org/>). Heatmap was drawn by R software.


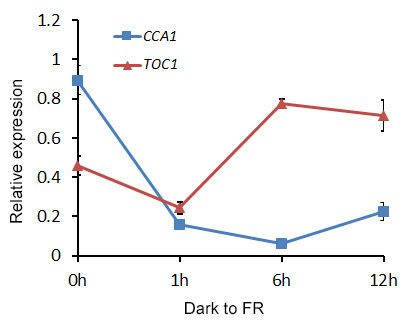


Supplementary Figure 4. Expression of *CCA1* and *TOC1* in response to FR irradiation. Seedlings grown in darkness for 4 d and then transferred to FR light for various time periods. Values are means ± SD; n=3.


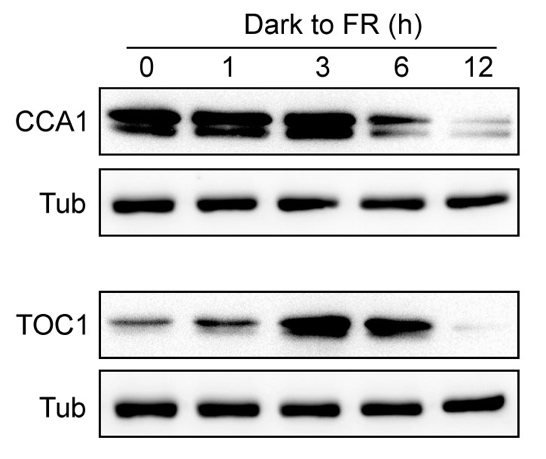


Supplementary Figure 5. ﻿Immunoblot assay of CCA1 and TOC1 protein accumulation in response to FR treatment. Four-day-old dark-grown *35S::FLAG-CCA1-HA* and *35S::FLAG-TOC1-HA* seedlings were exposed to far-red light, and collected at the indicated times. Anti-FLAG was used to detect CCA1 and TOC1 proteins. Tubulin was used as a loading control.


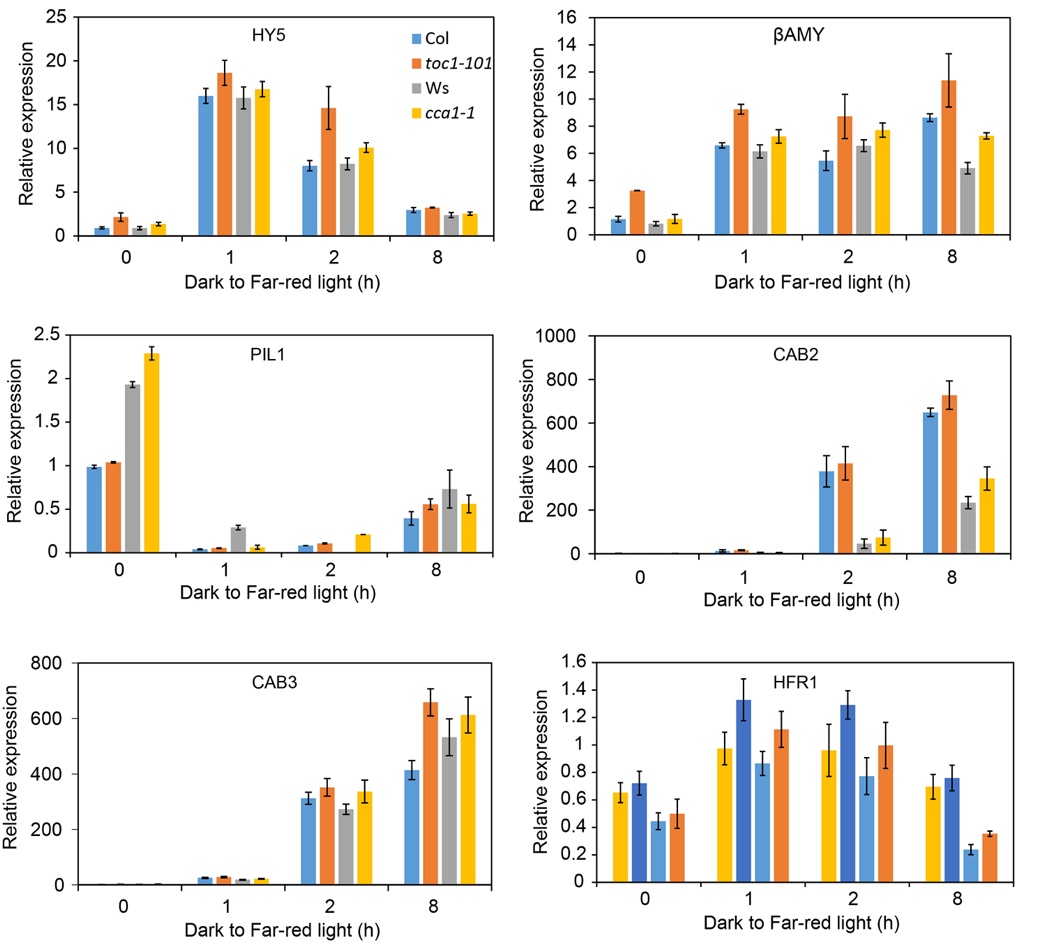


Supplementary Figure 6. Expression analysis of FR responsive genes in *toc1-101* and *cca1-1* mutants. qRT-PCR analysis of *HY5*, *βAMY*, *PIL1*, *CAB2*, *CAB3* and *HFR1* expression in *toc1-101* and its wild-type background (Col), *cca1-1* and its wild-type background (Ws). Seedlings were grown in darkness for 4 d and then transferred to FR light for various time periods. Values are means ± SD; n=3.


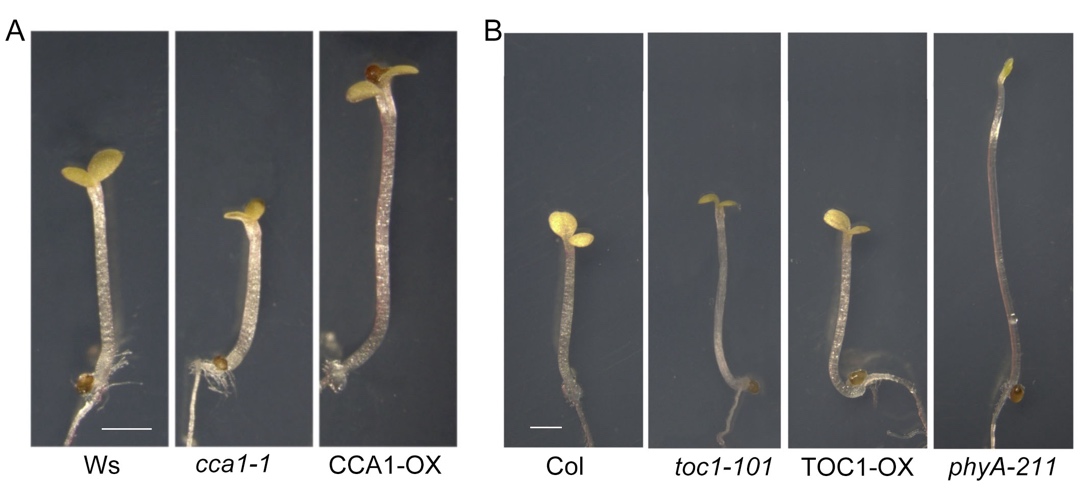


Supplementary Figure 7. Phenotypic analysis of hypocotyl growth under continuous FR light. ﻿Bar = 2mm.

Supplementary Table 1. Primers Used in This Study

| Primer | Usage | Sequence (5’ to 3’) |
| --- | --- | --- |
| CCA1 | qPCR | TCTGTGTCTGACGAGGGTCGAATT |
|  |  | ACTTTGCGGCAATACCTCTCTGG |
| TOC1 | qPCR | ATCTTCGCAGAATCCCTGTGATA |
|  |  | GCACCTAGCTTCAAGCACTTTACA |
| FHY1 | qPCR | GATGAAAGAGGAATCATCTGGA |
|  |  | AATCCTCTAAGTTCTGAGTCCCA |
| FHL | qPCR | ACCCAAGACTTTACTCACATGG |
|  |  | CACTCCACCTTGCAGAAGAA |
| TOC1 | qPCR | ATCTTCGCAGAATCCCTGTGATA |
|  |  | GCACCTAGCTTCAAGCACTTTACA |
| HY5 | qPCR | gtcccgctcttttcctctttatc |
|  |  | GTCGCTTGTTCCTGCATTTTTC |
| B-AMY | qPCR | TCGGGAAAACTAACTGGGGAACA |
|  |  | ACTCGCTATTCCATGTTCCGTCTCT |
| PP2a | qPCR | TAACGTGGCCAAAATGATGC |
|  |  | GTTCTCCACAACCGCTTGGT |
| TOC1 | Transient assay | CGATAGTACTGTCGACATGGATTTGAACGGTGAGTGTAAAG |
|  |  | TACCCTCGAGGTCGACAGTTCCCAAAGCATCATCC |
| CCA1 | Transient assay | CGATAGTACTGTCGACATGGAGACAAATTCGTCTGGAGAA |
|  |  | TACCCTCGAGGTCGACTGTGGAAGCTTGAGTTTCCA |
| FHY1pro | Y1H | ACTGAATTCGTCAGTAACATCTCCTAAGCAACG |
|  |  | ACTCTCGAGAGATCGCAGAGAGAGAGAGAGAG |
| FHLpro | Y1H | TATTGGATCGGAATTCTATAGTTTTTTGCCTACGTAGAAATGG |
|  |  | GAGCACATGCCTCGAGGATCTTAGAAGATTAATTTTCTTCCTCC |
